# *Culicoides* Midge Bites Modulate the Host Response and Impact on Bluetongue Virus Infection in Sheep

**DOI:** 10.1371/journal.pone.0083683

**Published:** 2014-01-08

**Authors:** Nonito Pages, Emmanuel Bréard, Céline Urien, Sandra Talavera, Cyril Viarouge, Cristina Lorca-Oro, Luc Jouneau, Bernard Charley, Stéphan Zientara, Albert Bensaid, David Solanes, Joan Pujols, Isabelle Schwartz-Cornil

**Affiliations:** 1 Centre de Recerca en Sanitat Animal CReSA, Universitat Autònoma de Barcelona – Institut de Recerca i Tecnologia Agroalimentaries, Bellaterra, Spain; 2 Virologie, Unité Mixte de Recherche UMR1161, Agence Nationale de Sécurité Sanitaire de l'Alimentation, de l'Environnement et du Travail – Institut National de la Recherche Agronomique – Ecole Nationale Vétérinaire d'Alfort, Maisons-Alfort, France; 3 Virologie et Immunologie Moléculaires, Unité de Recherche UR892, Institut National de la Recherche Agronomique, Jouy-en-Josas, France; Metabiota, United States of America

## Abstract

Many haematophagous insects produce factors that help their blood meal and coincidently favor pathogen transmission. However nothing is known about the ability of *Culicoides* midges to interfere with the infectivity of the viruses they transmit. Among these, Bluetongue Virus (BTV) induces a hemorrhagic fever- type disease and its recent emergence in Europe had a major economical impact. We observed that needle inoculation of BTV8 in the site of uninfected *C. nubeculosus* feeding reduced viraemia and clinical disease intensity compared to plain needle inoculation. The sheep that developed the highest local inflammatory reaction had the lowest viral load, suggesting that the inflammatory response to midge bites may participate in the individual sensitivity to BTV viraemia development. Conversely compared to needle inoculation, inoculation of BTV8 by infected *C. nubeculosus* bites promoted viraemia and clinical symptom expression, in association with delayed IFN- induced gene expression and retarded neutralizing antibody responses. The effects of uninfected and infected midge bites on BTV viraemia and on the host response indicate that BTV transmission by infected midges is the most reliable experimental method to study the physio-pathological events relevant to a natural infection and to pertinent vaccine evaluation in the target species. It also leads the way to identify the promoting viral infectivity factors of infected *Culicoides* in order to possibly develop new control strategies against BTV and other *Culicoides* transmitted viruses.

## Introduction

Several biting midges of the *Culicoides* genus (Diptera: Ceratopogonidae), that are among the smallest haematophagous insects, are transmission vectors of viruses affecting human and animals, such as the Oropouche virus in human [1], the African Horse Sickness and Equine Encephalosis viruses [2], the zoonotic human/swine Vesicular Stomatitis Virus [3] and three ruminant viruses with the Bluetongue virus [4], the Epizootic Haemorrhagic Disease virus [2] and the Schmallenberg virus [5]. Bluetongue virus (BTV) - the most studied virus transmitted by *Culicoides* midges so far - belongs to the *Orbivirus* genus of the *Reoviridae* family, and includes 26 serotypes [6] determined by the outer capsid VP2 and, to a lesser extent VP5 proteins [4]. Several BTV serotypes, mainly BTV8 and 1, have been recently introduced in Europe where they caused major economical damages in the cattle and sheep industry with loss of production and mortality and trade restrictions [4]. The clinical manifestations of Bluetongue (BT) can include fever, salivation, hyperaemia/oedema, mucosae ulcerations, and abortion [4]. BTV is transmitted by many *Culicoides* species, with dominant species that vary in different parts of the world, such as *C. imicola* in Africa, *C. sonorensis* in North America, *C. brevitarsis* in Australia and *Culicoides* of the so called Obsoletus group in Northern Europe [7].

Blood sucking arthropods are often more than just delivery systems for the pathogens they carry. Indeed the arthropod bite triggers vasoconstriction, the immediate onset of acute inflammation and of innate immune responses that may interfere on the pathogen transmission. The saliva of haematophagous arthropods contains a cocktail of pharmacologic proteins that modulate vascular constriction, blood coagulation, inflammation and immunity [8]. These compounds, mainly studied in the cases of ticks, sand flies, and mosquitoes, contribute to the success of the blood meal and can also promote pathogen infectivity [8]. Regarding mosquito- transmitted viral infections, West Nile Virus infection in mice was enhanced by co-administration of mosquito saliva [9]; West Nile Virus [10] or Cache-Valley Virus [11] infections were exacerbated when the viruses were inoculated in the skin area subsequently to the feeding of mosquitoes in mice. However no effect of mosquito bite was observed in the case of Saint Louis Encephalitis Virus and Western Equine Encephalitis Virus transmission in chicken [12]. Thus the effect of insect bites on infection may depend on the pathogen, on the insect type, and possibly on the vertebrate species.

*Culicoides* are “pool” feeders: they lacerate the skin to feed on the effusion into this injury, which includes blood, skin cells and lymph. Nothing is known about the effect of *Culicoides* bite on viral transmission, an information of importance for the development of control strategies. *Culicoides* secrete molecules such as vasodilatator [13], anticoagulant [14] and saliva components that are able to inhibit mouse lymphocyte response and nitric oxide production by macrophages [3]. The *Culicoides* bite provokes the recruitment of inflammatory cells that might be targets for viruses such as BTV [15]. In addition, BTV produced by insect cells *in vitro* seems more infectious for sheep than BTV produced by mammalian cells [16]. However, *Culicoides* saliva cleaves the BTV VP2 outer capsid protein, resulting in processed viruses that are less infectious for mammalian Baby Hamster Kidney cells compared to the native particles [17]. Thus *Culicoides* bites may positively or negatively affect BTV transmission by the secreted saliva components, the host response to the mechanical action of their mouth part in the skin and to the saliva, the viral processing by the insect cells in the salivary gland and the sub-anatomical site where the virus is inoculated. In order to evaluate whether and how *Culicoides* bites may modulate BTV transmission, we compared the extent of viraemia, clinical signs and host responses induced by 3 infection modes with a European isolate of BTV8: 1) simple intradermal needle inoculation; 2) intradermal needle inoculation in the feeding site of C. *nubeculosus* as done in the case of West Nile Virus [10] or Cache-Valley Virus [11], and 3) transmission by intra-thoracically- infected C. *nubeculosus* midges. *C. nubeculosus* was chosen as it is the only European *Culicoides species* that can be properly maintained in laboratory conditions using standardized techniques [18]. Moreover, *C. nubeculosus* supported BTV multiplication both after virus ingestion and inoculation, and infected midges were able to transmit the virus in laboratory conditions [19]. We performed the experiments in sheep, the real target of BTV, in order to evaluate the tri-partite insect/virus/vertebrate interactions in the pertinent host species. Previous studies of pathogen transmission by insects have used mice as a model system and have very rarely involved natural target species. These experiments revealed that *C. nubeculosus* bites have a dual effect on BTV viraemia and clinical BT disease, with an inhibitory effect of control bites on needle- transmission and an enhancing effect of infected bites compared to needle- transmission, associated to differentially modulated innate host responses.

## Materials and Methods

### Ethics statement

All experiments were performed in the BSL3 facilities of the Centre de Recerca en Sanitat Animal (CReSA, Spain) under the guidelines of the European Community (86/609) and were approved by the Committee on the Ethics of Animal Experiments of *Universitat Autònoma de Barcelona* (UAB) # 1425 and Animal Experimentation Commission of DAAM # 6330. Specifically, sheep were housed under temperature, humidity and light control with 4 stages trying to simulate the day light of the farms. Food, water and block of minerals were provided ad libitum. In the current experiment, symptoms were mild (<8 on a scale of 21), thus none of the animals required pain relief. Moreover, handling of animals was performed by experienced accredited workers, avoiding undue supplemental stress. Animals were euthanized with an overdose of sodium thiopental injected intravenously.

### Virus

The baby hamster kidney (BHK21) cell line used for virus growth and titration was grown in MEM supplemented with 5% FBS, 1% pyruvate, 1% non essential amino acids, and penicillin/streptomycin ( = culture medium). The wild-type field BTV8 strain was from the National Reference Laboratory collection (ANSES, Maisons-Alfort, France); it has been isolated from sheep blood in France in Ardennes in 2006, cultured on embryonated chicken eggs and then passaged only 3 times in BHK21 cells. To prepare BTV stocks, confluent BHK21 cell layers were infected and incubated at 37°C in 5% CO_2_ until the appearance of cytopathic effects (3 to 4 days post infection). Culture supernatants were collected and stored at −80°C. Fifty percent tissue culture infective doses (TCID_50_) were estimated by endpoint titration on BHK21 cells (method of Spearman-Karber) by using a previously described protocol [20]. The inoculation stock used in this study titrated 7.5×10^6^ TCID_50_/ml that represented 4.57×10^11^ BTV RNA copies/ml.

### BTV inoculation to midges and biting chambers

BTV8 (0.5–1 µl viral stock, 7.5×10^3^ TCID_50_) was inoculated intra-thoracically to *C. nubeculosus* females. Briefly, 2 or 3 day old females were slightly anesthetized using CO_2_ under a dissecting microscope. Using a glass microneedle and a manual microinjector, females were intra-thoracically inoculated with BTV8 inoculum through the suture between the epimeron and episterum with a volume that forces the cerci to slightly extend the tip of their wings (0.5–1 µl). Inoculated midges (n = 356 females) were sorted in 16 groups of 15–25 midges inside primary containers and maintained for an extrinsic incubation period of 6–7 days at 24±2°C, 75%RH, photoperiod 14 : 10 (light : dark) and feed *ad libitum* with 5% sucrose. To increase the number of blood engorged individuals while feeding on sheep in the pre-sensitization procedure, midges were starved for a period of 24 h and then fed on sheep. RNA was extracted from *Culicoides* right after the intra-thoracic inoculation (4 midges) and in the pools of midges after gorging on sheep and tested for content in BTV8 RNA copies (Table S1). The infectivity for mammalian cells of the *C. nubeculosus-* recovered BTV was confirmed on BHK-21 cells. However we could not get reproducible infectivity titers, possibly related to *Culicoides*- derived compounds interfering on BHK-21 receptivity.

### Sheep and experimental infection

Thirty two 6 month old Lacaune female sheep that had been checked to be BTV free and BTV seronegative were bought from Gregori Farm, Vilamajor d'Ager, Lleida (Spain). They had been treated with Deltametrine insecticide (Butox 7,5%; Intervet Schering-Plough AH) every four weeks from February to May and kept in protected area to avoid exposure to environmental midges. After a week acclimation in the CReSA BLS3 facilities (Bellaterra, Spain), the sheep were randomly allocated in 5 groups (Table S2): (1) the control group (4 sheep) was intradermally inoculated with culture medium; (2) the control bite group (4 sheep, CB group) was treated by application, on the right and left inguinal areas, of *Culicoides* biting chambers with uninfected midges for 30 minutes just prior to intradermal inoculation with culture medium in the bitten zone; (3) the intradermal group (8 sheep, ID group) was infected by the intradermal route with a total of 3.75×10^6^ TCID_50_ (2.28×10^11^ BTV RNA copies) per sheep; (4) the CB + ID group (8 sheep) consisted of sheep treated by application of *Culicoides* biting chambers with uninfected midges for 30 minutes followed by intradermal inoculation with a total of 3.75×10^6^ TCID_50_ BTV8 per sheep (2.28×10^11^ BTV RNA copies), in the feeding site of the *Culicoides*; (5) finally, the infected bite group (8 sheep, IB group) was infected by application of two *Culicoides* biting chambers for 30 minutes with intra-thoracically infected midges, simultaneously placed on the right and left inguinal areas; as discussed in the result section, the mean RNA copy numbers available in the *Culicoides* was 2.11±1.4×10^10^ per sheep. However the exact amount of BTV delivered to each sheep was not known in this group.

In the groups that were needle- injected, the inoculum (medium or virus) was given intradermally in the inguinal zones in 10 spots (50 µl each), with 5 spots on the right side and 5 spots on the left side. In the groups that were exposed to control or infected *Culicoides* midges, 2 biting chambers were used, one applied in the right inguinal area while the second in the left one. Biting chambers consisted on cylindrical polypropylene containers (55 mm diameter) sealed with fine mesh (1 mm wide). One of the sides was opened to introduce anesthetized *Culicoides* midges and after that immediately sealed.

### Sample collection, local inflammation monitoring and clinical scoring

EDTA-blood samples were collected daily during the first 9 days post inoculation (dpi) and at day 11, 15 and 21 dpi. Serum samples were collected at 0, 5, 8 15 and 21 dpi. Body temperature was recorded daily for 16 days. Clinical signs were recorded daily. The clinical score table for the BTV8 animal trial is shown Table S3 and was established based on previous reports [16], [21], [22]. An integrated clinical score was calculated from the area under the curve for each sheep from 6 to 17 dpi using the Prism 5.0 software; no clinical signs were expressed before 6 dpi. The local inflammatory reaction at the inoculation point on both inguinal sides was monitored each day from 0 to 7 dpi and graded from 0 to 3 based on the intensity and area size of the local hyperaemia (score 0 to 3 per inguinal side). The integrated inflammatory reaction score per sheep was calculated from the area under the curve over the 0 to 7 dpi period.

### RNA extraction

For RNA extraction of midges, midges were homogenized in 500 µl of PBS using microbeads- containing tubes and a Ribolysor apparatus (Bio-rad, Marne-la Coquette, France). After clarification by centrifugation (2 minutes, 1000 g), RNA was extracted using the Qiamp viral RNA kit (Qiagen).

Viral RNA was extracted from 100 µl whole blood using the Qiamp viral RNA kit. Cellular RNA was extracted from EDTA blood using Trizol-LS extraction. The extracted cellular RNA was checked for quality with an Agilent 2100 Bioanalyzer using RNA 6000 Nano Kits (Agilent Technologies).

### Viral RNA detection

Viral RNA detection was performed with a commercial pan-BTV rt-RT-PCR (ADIAVET® BTV Real-time A352, ADIAGENE, France) targeting the segment 10 of BTV and GAPDH as an internal control. The quantification was done using a viral dsRNA corresponding to the BTV8 segment 10; briefly, total RNA were extracted from an infected culture cell by trizol extraction and the viral dsRNA were remove from ssRNA by lithium chloride (liCl) precipitation [23]. After migration and gel purification from a 1% agarose gel, the ds segment 10 was quantified by spectrophotometer. The number of BTV RNA copies per ml of blood was calculated at 0 to 9, 11, 15 and 21 dpi. The area under the curve over the 21 day-period was calculated using the Prism 5.0 software. An area under the curve below 2 log10 corresponded to non-viraemic sheep.

### Sheep cytokine and IFN- induced gene mRNA detection

For quantitation of cytokine and IFN-induced gene expression, RNA (200 ng) was reverse transcribed using random primers and the Multiscribe reverse transcriptase (Applied Biosystem). Real-time PCR (qPCR) was carried out using 5 ng cDNA with 300 nM primers in a final reaction volume of 25 µl of 1× SYBR Green PCR Master Mix (Applied Biosystem). The primers used to amplify ovine cDNA were designed with the Primer Express software (v2.0) using publically available GenBank sequences (Table S4). PCR cycling conditions were 95°C for 10 min, linked to 40 cycles of 95°C for 15 sec and 60°C for 1 min. Real-time qPCR data were collected by the Mastercycler® ep realplex - Eppendorf system. The expression of the different genes (arbitrary units) compared to T0 and relatively to RPS24 (ribosomal protein 24) or GAPDH was calculated by the 2^−ΔΔCt^ method with the Realplex software.

### Serological responses to BTV

The collected sera were analyzed using a commercial BTV VP7 competitive ELISA kit (ID-VET, Montpellier, France) according to manufacturer's protocols. The development of neutralizing antibody response was assessed in BHK21 cells by serum neutralization with 50 TCID_50_ BTV8 as described [24]. All dilutions were performed in triplicates.

### Statistical analyses

The qPCR viral load, Mx1 and CXCL10 gene expressions, the temperature and clinical scores, and the antibody response measurements were analyzed for statistical differences between sheep groups using the non-parametric one-sided *Mann-Whitney U* for independent groups.

## Results

### Contrasted effects of uninfected and infected *C. nubeculosus* bites on induction of BTV viraemia and clinical disease

We set up our goal to evaluate the effect of uninfected and infected *Culicoides* bites on BTV infection in sheep. In order to test the effect of uninfected bites, BTV was inoculated intradermally in sheep within the feeding sites of uninfected *C. nubeculosus* (control bite + intradermal, CB + ID group), and within the skin of unbitten sheep (intradermal, ID group). The number of bites per sheep in the CB + ID group varied between 11 and 50 (data not shown). We used BTV8, the BTV serotype that spread in Europe in 2006–2008, at a dose per sheep of 3.75×10^6^ TCID 50 corresponding to 2.28×10^11^ BTV RNA copies that is similar to conditions that previously induced viraemia and clinical symptoms in sheep [16], [25]. For testing the effect of infected bites, the sheep skin was bitten by BTV8- intra-thoracically inoculated *C. nubeculosus* (between 15 and 49 bites per sheep, IB group, data not shown). The same BTV8 preparation was used to infect the sheep and the *C. nubeculosus*. Four days post intra-thoracic inoculation, the mean number of BTV8 RNA copies reached 1.2×10^9^/midge, whereas the initial quantity of BTV8 RNA copies detected after inoculation was 2.43×10^7^/midge (Table S1), indicating that viral replication occurred in *C. nubeculosus* after intra-thoracic inoculation, in the same order of magnitude as previously reported for C. *soronensis* [26]. The BTV8 RNA load in the total engorged *Culicoides* per sheep varied between 6×10^9^ and 4.95×10^10^ BTV8 RNA copies per exposed sheep (mean 2.11±1.4×10^10^, Table S1). Therefore, the total quantity of BTV8 RNA available in the *C. nubeculosus* was about 10 times lower than in the intradermally- injected BTV8 inoculum.

**Figure 1:**
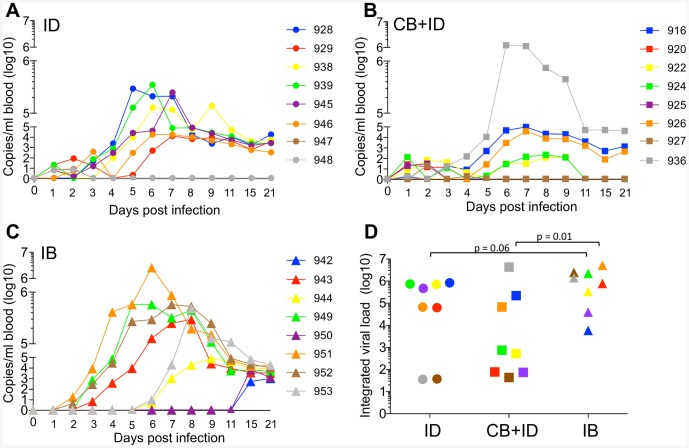
Measurement of viraemia in needle and *C. nubeculosous*- infected sheep. BTV viral RNA was detected in blood by qRT-PCR at 0–9, 11, 15, 21 dpi. The number of BTV copies per ml of blood is reported for each sheep with a specific color and symbol shape. The 3 infected group are represented by a distinct symbol, i.e. a circle for the intradermal needle- inoculation group (ID, **A**), a square for the control *C.* bite + intradermal needle- inoculation group (CB + ID, **B**), and triangle for the infected C. bite group (IB, **C**). In **D**, the viral load over the 21- day monitoring period was integrated for each sheep using a calculation of area under the curve (color and shape symbol as in A, B, C). Statistical comparisons between 2 groups were done with *a Mann-Whitney U* test and the p values are reported to show significance and major tendencies.

The BTV8 RNA load in blood peaked between 5 and 7 dpi in the ID and CB + ID groups (Fig. 1A and B), whereas the peak time was more variable in the IB group (Fig. 1C), occurring between 6 and 15 dpi. When the BTV8 RNA load per sheep was integrated for the whole monitoring period (Fig. 1D), only 3 out of 8 sheep in the CB + ID group presented an integrated viral load above 3 log10 copies/per ml, whereas 6 out of 8 sheep in the ID group did. This indicates that uninfected *C. nubeculosus* bites rather inhibited BTV viraemia onset. Conversely, despite the fact that the sheep from the IB group likely received far less BTV than the CB + ID and ID sheep, all IB sheep showed integrated viral loads above 3 log10, with 4 out of 8 sheep presenting high loads that were above 6 log10, a value not reached in the ID group (Fig. 1D). Furthermore the viral load values tended to be higher in the IB group compared to the ID group (p = 0.06). In addition, 3 sheep from the IB group developed rapid viraemia that was detected at day 3, earlier than for any sheep of the other groups (Fig. 1C). Control sheep groups (injected with medium after exposure to uninfected *Culicoides* bites (control CB) or unexposed (control)) did not present any detectable BTV RNA in their blood. Thus we show that infected bites of *C. nubeculosus* transmit BTV infection to sheep, as previously shown for *C. soronensis* in experimental conditions [26], and even appear to promote BTV transmission and high viraemia compared to needle transmission. Conversely uninfected bites combined to needle transmission rather tended to reduce BTV viraemia compared to plain needle transmission.

**Figure 2:**
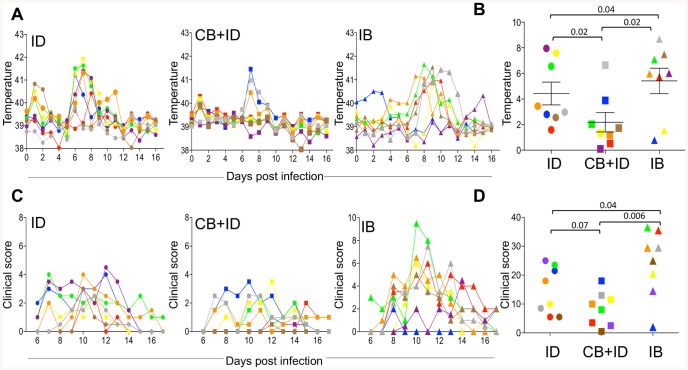
Body temperature and clinical scoring in needle and *C. nubeculosous-* infected sheep. A. Body temperature was monitored for each sheep over 17 days. **B.** The increase in temperature over time was calculated relatively to day 0 and the peak area under the curve was calculated for each sheep. **C.** A clinical score was evaluated for each sheep and reported from 6 to 17 dpi in B. **D.** The integrated area under the curve of the clinical scores from 6 to 17 dpi was calculated for each sheep. The same color and shape are used for each sheep as in Fig. 1. Statistical comparisons between 2 groups were done with *a Mann-Whitney U* test and the p values are reported to show significance and major tendencies.

The body temperature monitoring indicated that 5 sheep out of 8 in the ID group and 6 sheep out of 8 in the CB + ID group presented an early temperature rise at 1 dpi, that was not observed in any sheep of the IB group (Fig. 2A), nor in the control sheep. In the ID group, a clear temperature rise, that corresponded to an integrated increase of temperature >2°C, was observed between 5 and 9 dpi in 7 out of 8 sheep (Fig. 2A and B). Conversely only 3 sheep in the CB + ID group presented a body temperature rise (Fig. 2A and B). In the IB group, the temperature rise was observed in 6 of 8 sheep, from 6–11 dpi, starting a slightly later but lasting longer than in the ID group (Fig. 2A and B). No temperature rise was observed in the control sheep. Clinical signs were observed starting from day 6 in all infected groups (Fig. 2C). The integrated clinical scores tended to be lower in the CB+ID group and were higher in the IB group compared to the scores in the ID group (p = 0.07 and 0.04 respectively, Fig. 2D). The control sheep showed neither symptoms nor body temperature rise (data not shown). There was a significant correlation between the RNA viral load measured in Fig. 1 and the clinical symptom scores reported in Fig. 2 (Figure S1). Altogether, these data show that infected bite transmission tended to increase the clinical symptoms severity of BTV infection, compared to needle transmission. Conversely, uninfected bites reduced the clinical disease and temperature rise induced by needle transmission.

### The intensity of local inflammatory reaction in uninfected *C. nubeculosus* bitten sheep is associated to low viral load

**Figure 3:**
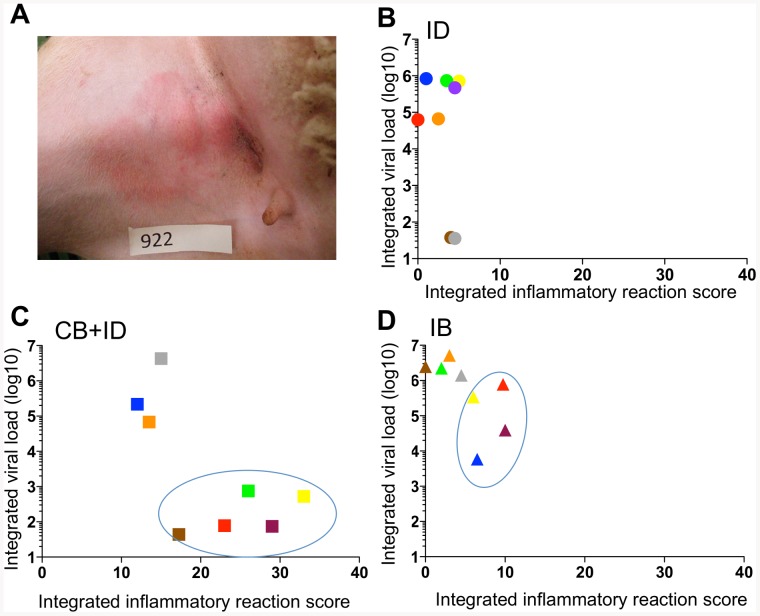
An elevated local inflammatory reaction appears associated to lower viraemia in *C. nubeculosous-* bitten sheep. **A.** A local inflammation at the inoculation point was observed especially in the CB + ID group, with an example of sheep 922 (score = 3). **B, C, D.** The local inflammation of the inoculation points was monitored from 0 to 7 dpi (the scores from the right and left inguinal sides were added) and the calculated area under the curve for each sheep is reported with the same color and shape symbol as in Fig. 1. B: ID group, C: CB + ID group, D: IB group. The sheep with higher inflammation and lower viral loads in the CB + ID and IB groups are circled.

During the course of the clinical monitoring, we observed that many sheep developed inflammatory reactions at the inoculation points (Fig. S2, Fig. 3A). The highest global local reaction, measured by the integrated local inflammatory score over the 0–7 dpi period, was encountered in the CB + ID group, and the intensity of the local reaction was independent of the bite numbers (Fig. S2A and B). The ID and IB groups also presented significant local inflammatory reactions compared to the control group inoculated with medium (Fig. S2A). Interestingly in the CB + ID group, the sheep that showed the highest local reaction (integrated inflammatory score above 17) presented the lowest viral loads, below 3 log10 copies/ml (Fig. 3C). Even in the IB group in which the local inflammatory reaction was weaker than in the CB + ID group (Fig. S2A), the sheep with the lowest inflammatory reaction (integrated inflammatory score below 5) had the highest viral loads, reaching >6 log10 copies/ml (Fig. 3D). In the ID group, the intensity of local inflammation was very weak and could not be related to the viraemia level. These clinical observation data suggest that the uninfected *C. nubeculosus* plus the intradermal BTV8 injection potentiate each other to trigger strong local inflammation, preventing BTV viraemia. These data also lead to propose the hypothesis that the local inflammation, triggered by *Culicoides* skin laceration, may have a negative effect on BTV viraemia onset.

### The systemic IFN- induced gene response is delayed in sheep bitten by infected C. *nubeculosus* compared to needle- infected sheep

**Figure 4:**
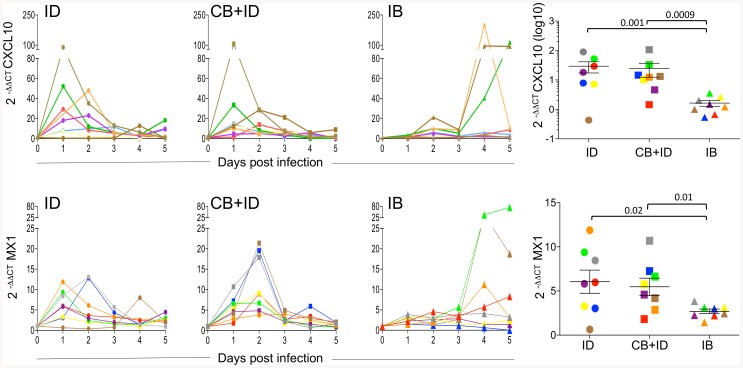
IFN- induced gene expression in blood cells is delayed in *C. nubeculosous-* infected sheep. Total RNA was extracted from blood cells of each sheep from 0–5 dpi. qPCR was performed on reverse transcribed RNA using the primers for CXCL10 and MX1 genes detailed in Table S3. The 2^−**ΔΔ**CT^ method was used to calculate the cytokine gene expression relatively the T0 time point level using the ribosomal RPS24 RNA as an internal control. The blood cells from control and control CB group did not show any induction of CXCL10 and MX1 gene expression during the observation period (not shown). The same color and shape symbol are used as in Fig. 1. Statistical comparisons between 2 groups were done with *a Mann-Whitney U* test and the p values are reported to show significance.

Expression of several innate immunity genes was detected in the blood cells of the control and infected sheep at 0–5 dpi (Fig. S3). There was no consistent induction of IL-1β, TNFα, XCL1, and CCL5 mRNA in infected versus control sheep. In the CB + ID group, the IL-8 mRNA expression was enhanced in sheep 920 and 925 (8 fold) and the IL-6 mRNA expression was enhanced in sheep 922 (5 fold), these sheep showing low to no viraemia and also high local inflammatory reaction; these cytokine genes were not induced to the same extent in the other sheep (Fig. S3). Sheep 953 (IB group) appeared as an over-reacting sheep, as it was the only sheep showing upregulation of most cytokine genes in addition to high fever. Interestingly, 2 IFN- induced gene mRNA, CXCL10 and MX1, were augmented at 1 or 2 dpi compared to day 0 in the 2 needle- inoculated groups (ID and CB + ID, Fig. 4) whereas in the IB group, the CXCL10 and MX1 mRNA levels were not augmented at early time points but raised at day 4 in sheep 951, 949 and 952 after the onset of viraemia. The increase in CXCL10 at day one was significantly higher in the ID and CB + ID groups than in the IB group (p = 0.001 and p = 0.0009 respectively). The increase in MX1 at day 1 was significantly higher in the ID and CB + ID groups than in the IB group (p = 0.02 and p = 0.01, respectively). Uninfected *C. nubeculosus* bite did not affect IFN- induced gene induction (data not shown). Unfortunately, type I IFN could not be detected in the serum of the infected sheep by using a biological assay, possibly due to too low amounts or inappropriate detection time points. These data indicate that IFN- induced gene induction is delayed when BTV8 is transmitted by infected *Culicoides* compared to needle transmission.

### The humoral anti-BTV response is delayed in sheep bitten by infected *C. nubeculosus* compared to needle- infected sheep

**Figure 5:**
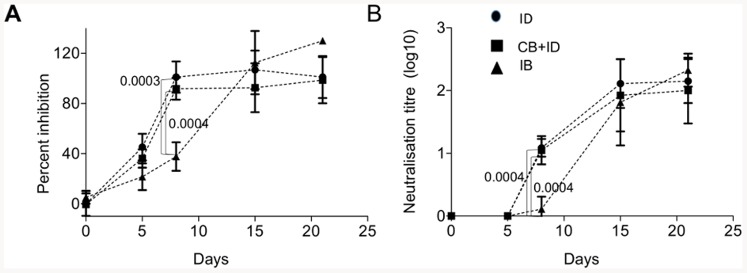
Anti-VP7 and neutralizing antibody response in serum is delayed in *C. nubeculosous-* infected sheep. **A.** Anti-VP7 antibodies were detected by competitive ELISA at 0, 5, 8, 15 and 21 dpi. **B.** Neutralizing antibodies were assessed at 0, 5, 8, 15 and 21 dpi in BHK21 cells by serum neutralization with 50 TCID_50_ BTV8 (triplicates per sheep and per day). Statistical differences between groups were calculated by *a Mann-Whitney U* test on each day and the p values are reported for day 8.

The anti-BTV antibody response in sera was assessed by anti-VP7 antibody detection using competition ELISA and by neutralizing antibody titration (Fig. 5). Anti-VP7 antibodies were detected as soon as by 5 dpi in the needle- infected sheep and plateaued at 8 dpi in these 2 groups, independently of the viraemia level. Conversely the anti-VP7 serological response was barely detectable at 8 dpi in the IB group and reached plateau values by 15 dpi. The neutralizing antibodies were already detectable at 8 dpi in all the needle- inoculated groups whereas it was not detectable in the IB group. Similar levels of neutralizing antibodies were reached at 15 dpi in all infected groups, independently of the viraemia level. These serological analyses show that the adaptive response to BTV occurred later in the IB group than in the ID and CB + ID groups.

Altogether our results suggest that the systemic innate and adaptive responses to BTV are delayed when BTV is transmitted by infected *C. nubeculosus* bites compared to needle administration, corresponding to higher viremia and more severe clinical disease.

## Discussion

We showed here that *C. nubeculosus* bites impacted on the infection of sheep by BTV. Uninfected bites prevented the onset of viraemia and occurrence of symptoms in the majority of the sheep that were needle- inoculated with BTV8, possibly related to the intensity of local inflammatory response. Conversely infected *C. nubeculosus* bites promoted viraemia and disease compared to needle transmission. Both systemic innate and adaptive host responses were delayed in the sheep exposed to infected *C. nubeculosus* bites compared to the needle- inoculated sheep. These data suggest that infected *Culicoides* bites are the optimal way for BTV transmission, by avoiding the development of an early host defense responses.

Inoculation of BTV8 in the *Culicoides* bitten sites rather suppressed the development of viraemia and disease in most sheep, in contrast with Cache Valley virus and West Nile virus administration in the feeding site of mosquitoe that promoted viral infection [10], [11]. *Culicoides* lacerate the skin and trigger local inflammation that was further amplified by the subsequent needle transmission of the virus (Fig. S2A). In this certainly artificial infection method, we observed that the sheep with the highest local inflammatory response had the lowest viral RNA load in the blood, indicating that the local inflammation may prevent systemic infection, viraemia and disease. In addition, in some of these low viraemic sheep, the IL-6 and IL-8 gene expression was upregulated at early time points in the blood cells, suggesting occurrence of systemic inflammatory response. It would have been interesting to test the cytokine response within the skin lesions but we favored avoiding possible artifacts induced by taking biopsies. Besides, the sheep of the CB + ID group did all develop anti-VP7 and neutralizing antibody responses, suggesting that sufficient viral amount was detected by the host immune system to trigger high humoral response. It is possible that the local inflammation confined BTV8 to the skin, and prevented dissemination to the blood stream while still allowing immune response onset. The inhibitory action of the control *C. nubeculosus* bite may be explained by the composition of the uninfected *Culicoides* saliva that may trigger inflammation, further potentiated by the intradermal BTV inoculum. However collection of sufficient saliva from this small insect was not feasible in our experimental set up for directly evaluating the effect of the saliva *in vivo*. It remains possible that the local inflammation induced by *C. nubeculosus* corresponds to a delayed type hypersensitivity reaction (DTH), resulting from a previous exposure of the sheep *to Culicoides* bites before the experiment in BSL3. Indeed in the case of *Leishmania* transmission by sandflies, DTH to insect salivary components was shown to be detrimental to *Leishmania* establishment [27]. In the case of *Culicoides*, DTH reactions have been detected in horses [28], but not in ruminants to our knowledge. In our setting, cautions were taken to avoid exposure to midges during previous housing (insecticide treatment), but midge antigen- directed DTH can not be formally ruled out. In any event, the extent of the local inflammation triggered by uninfected *C. nubeculosus* bites varied between the different sheep and may be brought as an hypothesis for the differential individual receptivity to BTV viraemia. Finally in the IB group, there was also a trend for lower viral load in the sheep with the highest local inflammation, suggesting that individuals with high inflammatory response to *Culicoides* bites may be less susceptible to development of BTV viraemia.

The high efficacy of infected *C. nubeculosus* compared to needle administration to promote high viraemia and more pronounced clinical symptoms is striking. Many factors differ between needle and insect administration that can interfere on each other and promote viral transmission. BTV processed by the insect cells and further modified in the saliva may present biochemical alterations compared to BTV produced in mammalian cells *in vitro*, favoring infection of the sheep. Indeed the VP2 protein from 3 different BTV strains was shown to be cleaved by treatment with saliva from adult *C. soronensis in vitro* [17]. The ratio of defective particles versus infectious particles may be different between the virus produced by whole insect and mammalian cell cultures, thus affecting the balance and kinetics of immunity and infection. As uninfected *C. nubeculosus* displayed an inhibitory effect on viral transmission, it remains possible that uninfected and infected *Culicoides* saliva have different composition. Indeed the salivary gland function and virus transmission efficiency changed during the course of WNV infection due to pathological changes in the mosquito [29], resulting in a differential salivary gland transcript profile [30]. In that scenario, infected *C. nubeculosus* saliva may modulate the host response, promote the observed delay in IFN- gene and antibody responses and prevent the early peak of body temperature (Fig. 2, 4 and 5). Indeed in the case of vesicular stomatitis New Jersey virus [31], insect saliva was shown to modulate type I IFN production and to affect host resistance to the virus. Finally the mode of virus inoculation by *Culicoides* may be favorable to hit or recruit the best cells for viral transmission and dissemination.

The viral doses received by the needle- and infected *Culicoides-* inoculated sheep were most probably very different. It is generally believed that the higher the dose, the shorter the incubation period and the time from inoculation to onset of viraemia and clinical signs [32], [33], [34]. In the case of BTV infection in cattle and sheep, the size of the inoculum did not seem to influence the kinetics of viraemia [35], [36], [37]. We used here a high dose of virus for challenge, representing a reliable challenge, as done previously in other studies [16], [25]. Whereas it was expected that the lower viral inoculum delivered by the infected *Culicoides* would have been an handicap for efficient infection, it is possible that on the contrary, the high viral load in the needle- inoculum may have induced a rapid counteracting immunity that interfered with the infection efficacy. A escalating dose of BTV inoculum in parallel with evaluation of the host immunity response may help to solve this issue.

Altogether our study indicates that infected *C. nubeculosus* are more efficient than the needle- inoculation of high viral dose of BTV to induce high viraemia and BT disease in sheep. *C. nubeculosus* bites had a dual effect in our experiment, with infected bites promoting infection, and uninfected bites inhibiting infection by needle- inoculation. These results should be extended to other *Culicoides* species in order to be generalized. However our results unravel that needle- inoculation of high viral load induce the rapid onset of systemic innate and adaptive immune responses, whereby probably inhibiting the viral infection, and modifying the physio-pathological steps of BTV infection occurring in the natural infection. Uninfected bites appeared to enhance the local inflammation and further impede the infection by needle- inoculated BTV. Comparatively, infected *Culicoides* bites induced delayed immune responses that were detected after the onset of viraemia. The factors implicated in the efficacy of viral transmission by infected *Culicoides*, that are possibly related to the insect effect on the virus and/or on the host, remain to be identified. The understanding of the midge/host/virus interaction will result in the development of pertinent and novel control strategies. Indeed the insect could be targeted by vaccines, such as in the case of the SAP1 protein of sand fly that is considered as a promising target against their pathogen transmission [8]. In addition, inoculation of BTV by infected *Culicoides* stands as an optimal and reliable way to evaluate the physiopathological mechanisms of BTV infection and to test BTV targeted vaccine strategies, that may pertain to the other viruses such as Schmallenberg and African Horse Sickness viruses that are transmitted by *Culicoides* midges.

## Supporting Information

Figure S1 Correlation between clinical scores and viral loads across groups. The integrated clinical scores and the integrated viral loads were plotted for each sheep and the correlation between the X/Y value was found significant (Pearson r = 0.5, R square = 0.25, p-value = 0.0059). The same colors and shape symbols corresponding to each sheep were used as in Fig. 1.
(TIF)

Figure S2 Local inflammatory reaction after needle and *C*. transmission of BTV. **A.** The local inflammatory reaction score over the 0–7 dpi period is reported for the different sheep groups. Statistical comparisons between 2 groups were done with *a Mann-Whitney U* test and the p values are reported to show significant differences. **B.** The local inflammatory reaction score of each sheep of the CB+ID was plotted against the number of initial bites and the graph shows lack of correlation.
(TIF)

Figure S3 qPCR detection of cytokine gene expression in sheep blood cell RNA. **3A**: TNFα, IL-6, IL-1β mRNA. **3B**: IL-8, CCL5, XCL1. Total RNA was extracted from blood cells of each sheep from 0–5 day dpi. qPCR was performed on reverse transcribed RNA using the primers for cytokine genes detailed in Table S3. The 2^−ΔΔCT^ method was used to calculate the cytokine gene expression relatively the T0 time point level using the ribosomal RPS24 RNA or GAPDH as an internal control. The blood cells from control and control CB group did not show any induction of cytokine gene expression during the observation period. The same colors and shape symbols corresponding to each sheep were used as in Fig. 1.
(PDF)

Table S1 BTV RNA copy numbers in infected C. nubeculosus. The BTV RNA copy numbers in pools of infected C. nubeculosus were assessed by qPCR as described in the material and method section.
(DOC)

Table S2 Experimental design. The quantities of BTV administered to the different experimental groups of sheep are reported as TCID50 and RNA copy numbers.
(DOC)

Table S3 Clinical scores in BTV trial from 3–17 dpi. The severity of each listed symptom was graded from 0 to 3. The sum of the different symptom scores provided the clinical score per day for each sheep.
(DOC)

Table S4 Primers used to detect expression of sheep genes in blood cells (qPCR). Primers for the sheep genes were designed by the Primer Express software and provided >95% amplification efficiency.
(DOC)
