# Supplementary figures and images for: Culicoides Midge Bites Modulate the Host Response and Impact on Bluetongue Virus Infection in Sheep
Source: PLoS One. 2014 Jan 8;9(1):e83683. doi: 10.1371/journal.pone.0083683 (PMC3885445; doi:10.1371/journal.pone.0083683)

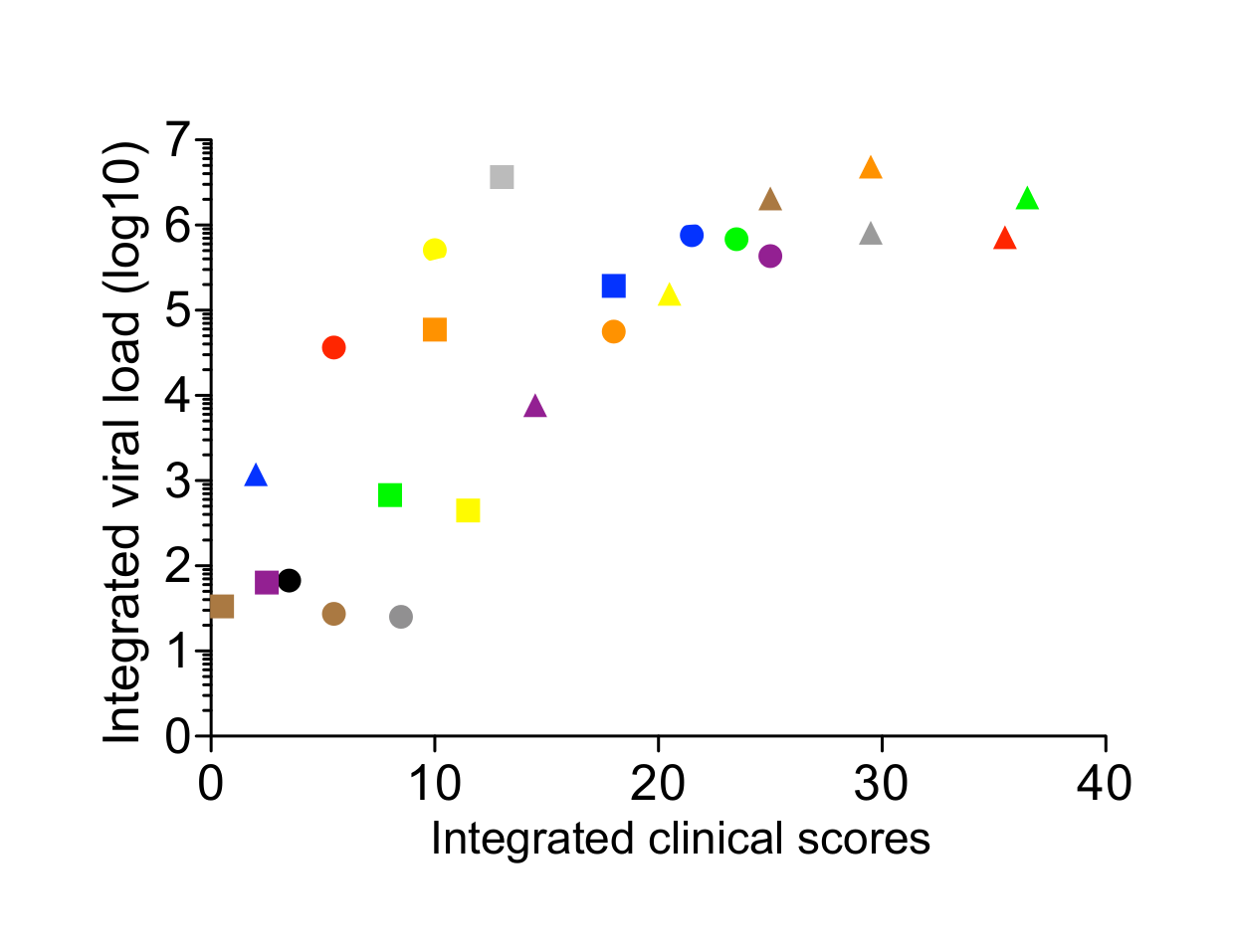

Supplement: Figure S1 — Correlation between clinical scores and viral loads across groups. The integrated clinical scores and the integrated viral loads were plotted for each sheep and the correlation between the X/Y value was found significant (Pearson r = 0.5, R square = 0.25, p-value = 0.0059). The same colors and shape symbols corresponding to each sheep were used as in Fig. 1. (TIF) [file pone.0083683.s001.tif]

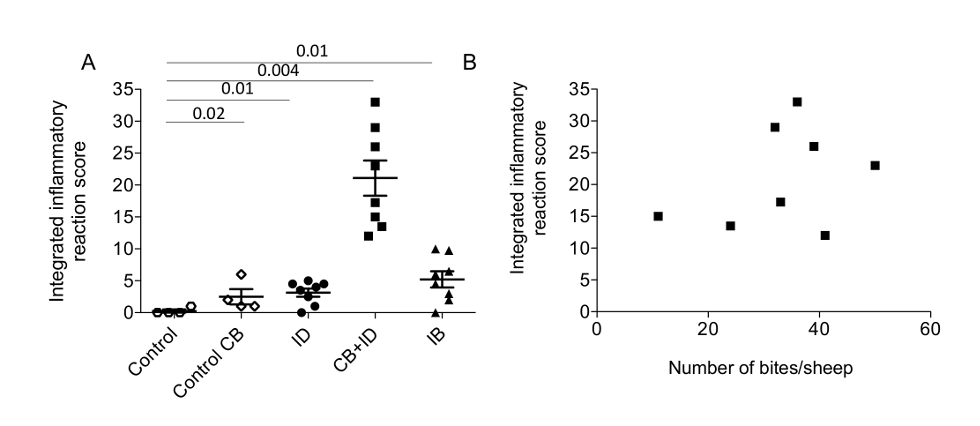

Supplement: Figure S2 — Local inflammatory reaction after needle and C. transmission of BTV. A. The local inflammatory reaction score over the 0–7 dpi period is reported for the different sheep groups. Statistical comparisons between 2 groups were done with a Mann-Whitney U test and the p values are reported to show significant differences. B. The local inflammatory reaction score of each sheep of the CB+ID was plotted against the number of initial bites and the graph shows lack of correlation. (TIF) [file pone.0083683.s002.tif]

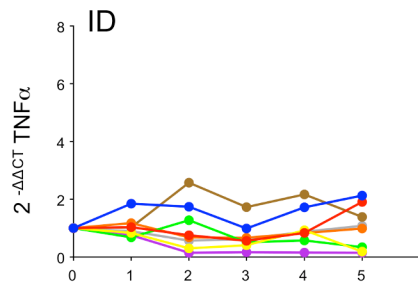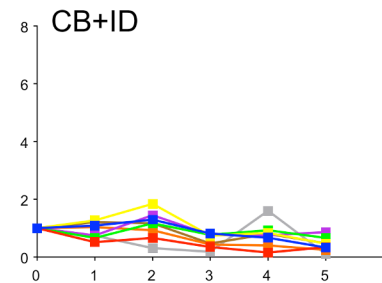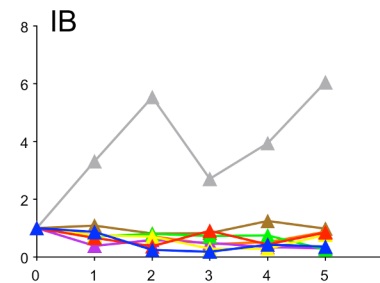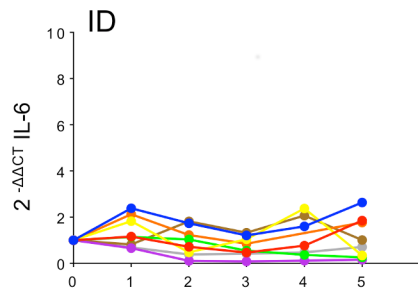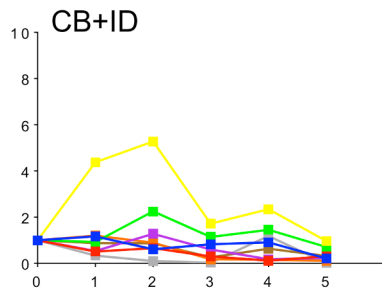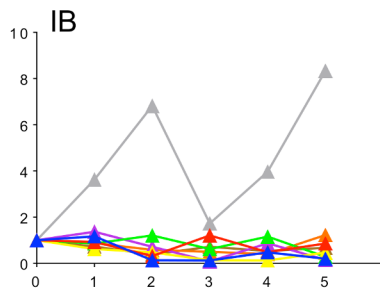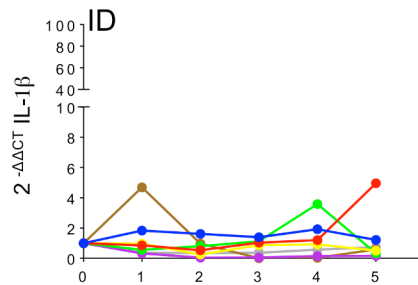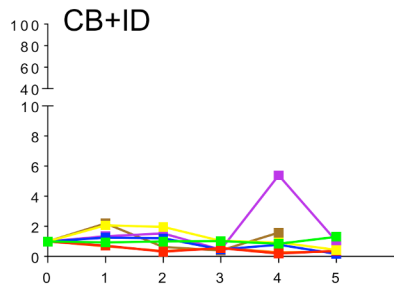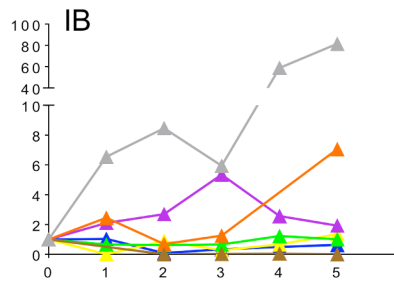

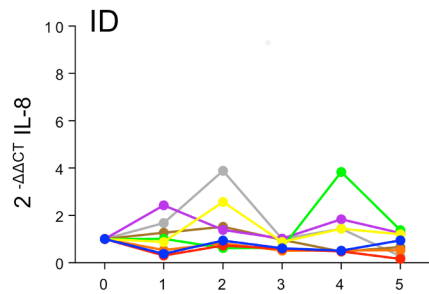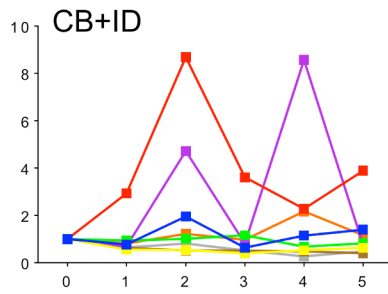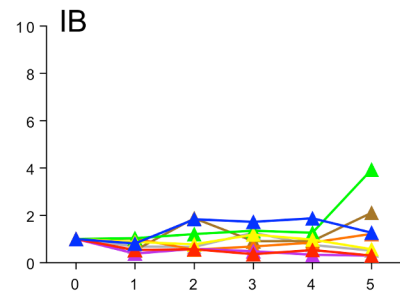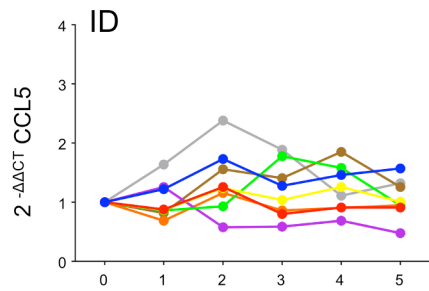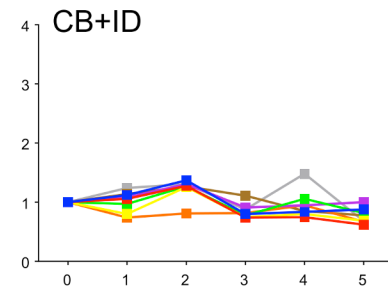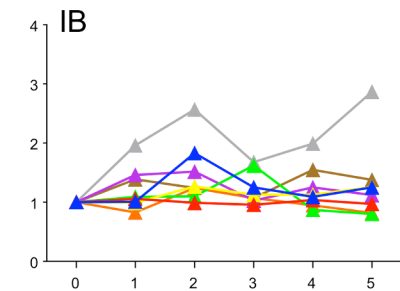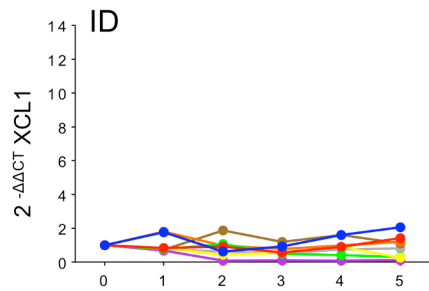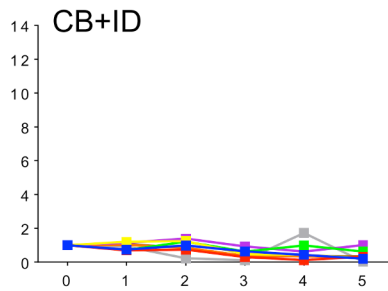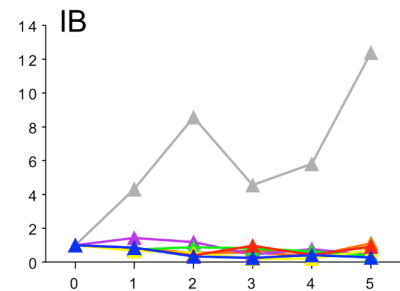

Supplement: Figure S3 — qPCR detection of cytokine gene expression in sheep blood cell RNA. 3A: TNFα, IL-6, IL-1β mRNA. 3B: IL-8, CCL5, XCL1. Total RNA was extracted from blood cells of each sheep from 0–5 day dpi. qPCR was performed on reverse transcribed RNA using the primers for cytokine genes detailed in Table S3. The 2−ΔΔCT method was used to calculate the cytokine gene expression relatively the T0 time point level using the ribosomal RPS24 RNA or GAPDH as an internal control. The blood cells from control and control CB group did not show any induction of cytokine gene expression during the observation period. The same colors and shape symbols corresponding to each sheep were used as in Fig. 1. (PDF) [file pone.0083683.s003.pdf]
