# Supplementary material for: Culicoides Midge Bites Modulate the Host Response and Impact on Bluetongue Virus Infection in Sheep
Source: PLoS One. 2014 Jan 8;9(1):e83683. doi: 10.1371/journal.pone.0083683 (PMC3885445; doi:10.1371/journal.pone.0083683)
Supplement: Table S1 — BTV RNA copy numbers in infected C. nubeculosus. The BTV RNA copy numbers in pools of infected C. nubeculosus were assessed by qPCR as described in the material and method section. (DOC) [file pone.0083683.s004.doc]

**Table S1. BTV RNA copy numbers in infected *C. nubeculosus***

| **Sheep #** | **Engorged midges** | **Non engorged midges** | **BTV RNA copy numbers in engorged midges per sheep** | **Mean BTV RNA copy numbers per engorged midge** |
| --- | --- | --- | --- | --- |
| Mean initial BTV RNA copies at T0 in inoculated midges (4) = 2.43 X 10^7^ | | | | |
| 942 | 18 | 13 | 1.94 X 10^10^ | 1.08 x 10^9^ |
| 943 | 16 | 15 | 6.45 X 10^9^ | 4.03 X 10^8^ |
| 944 | 25 | 4 | 3.06 X 10^10^ | 1.22 X 10^9^ |
| 949 | 10 | 7 | 9.83 X 10^9^ | 9.83 X 10^8^ |
| 950 | 15 | 11 | 4.95 X 10^10^ | 3.29 X 10^9^ |
| 951 | 15 | 10 | 8.24 X 10^9^ | 5.49 X 10^8^ |
| 952 | 17 | 9 | 6 X 10^9^ | 3.54 X 10^8^ |
| 953 | 23 | 11 | 3.92 X 10^10^ | 1.70 X 10^9^ |
| Mean ± SEM | 17.37 ± 3.46 | 10 ± 2.5 | 2.11 10^10^ ± 1.4 10^10^ | 1.20 X 10^9^ |
