# Supplementary material for: Culicoides Midge Bites Modulate the Host Response and Impact on Bluetongue Virus Infection in Sheep
Source: PLoS One. 2014 Jan 8;9(1):e83683. doi: 10.1371/journal.pone.0083683 (PMC3885445; doi:10.1371/journal.pone.0083683)
Supplement: Table S2 — Experimental design. The quantities of BTV administered to the different experimental groups of sheep are reported as TCID50 and RNA copy numbers. (DOC) [file pone.0083683.s005.doc]

**Table S2**. Experimental design

| Experimental condition | Groups | | | | |
| --- | --- | --- | --- | --- | --- |
|  | Control | Control CB | ID | CB + ID | IB |
| Number of sheep | 4 | 4 | 8 | 8 | 8 |
| Treatments | Medium ID | Uninfected *C. nubeculosus* + medium ID | BTV8 ID | Uninfected *C. nubeculosus* + BTV8 ID | BTV8 infected *C. nubeculosus* |
| BTV TCID50 |  |  | 3.75 x 10^6^ | 3.75 x 10^6^ | Could not be determined |
| BTV in RNA copy number |  |  | 2.28 X 10^11^ | 2.28 X 10^11^ | From 6 X 10^9^ to 4.95 X 10^10^ in the infected *Culicoides pool* per sheep |
