# Supplementary material for: Culicoides Midge Bites Modulate the Host Response and Impact on Bluetongue Virus Infection in Sheep
Source: PLoS One. 2014 Jan 8;9(1):e83683. doi: 10.1371/journal.pone.0083683 (PMC3885445; doi:10.1371/journal.pone.0083683)
Supplement: Table S3 — Clinical scores in BTV trial from 3–17 dpi. The severity of each listed symptom was graded from 0 to 3. The sum of the different symptom scores provided the clinical score per day for each sheep. (DOC) [file pone.0083683.s006.doc]

**Table S3.** **Clinical scores in BTV trial from 3-17 dpi.**

| Criteria | Signs and lesions | Scores |
| --- | --- | --- |
| Behavior | slowing movement, depression, lethargy, dullness ataxia, recumbence | 0 to 3 |
|  | decreased appetite, anorexia | 0 to 2 |
| Respiration | accelerated breathing, dyspnea | 0 to 2 |
|  | cough: occasional, repeated | 0 to 2 |
| Foot | coronitis, lameness/stiffness | 0 to 3 |
| Head lesions | face (mouth, lips, nose, tongue): congestion, edema, ulcers | 0 to 3 |
|  | nose : rhinitis, nasal discharge, mucous, purulent | 0 to 3 |
|  | eyes : slight reddening, palpebral edema, conjunctivitis, white haze | 0 to 3 |
| TOTAL | | < 21 |
