# Supplementary material for: Culicoides Midge Bites Modulate the Host Response and Impact on Bluetongue Virus Infection in Sheep
Source: PLoS One. 2014 Jan 8;9(1):e83683. doi: 10.1371/journal.pone.0083683 (PMC3885445; doi:10.1371/journal.pone.0083683)
Supplement: Table S4 — Primers used to detect expression of sheep genes in blood cells (qPCR). Primers for the sheep genes were designed by the Primer Express software and provided >95% amplification efficiency. (DOC) [file pone.0083683.s007.doc]

**Table S4. Primers used to detect expression of sheep genes in blood cells (qPCR).**

| Primer name  F forward, R reverse | Sequence |
| --- | --- |
| IL-1β-F | GCAGATTTCTCACAAGCTCTACAACA |
| IL-β-R | GCTTCTCCATGGCCACGAT |
| IL-6-F | GCTGCTCCTGGTGATGACTTC |
| IL-6-R | GGTGGTGTCATTTTTGAAATCTTCT |
| MX1-F | TGCGCATGGCTCAGGAT |
| MX1-R | CCAGATCGGGCTTTGTCAAG |
| IL-8-F | TTCCAAGCTGGCTGTTGCTCTCTT |
| IL-8-R | GCATTGGCATCGAAGTTCTGTACTC |
| TNFα-F | CAAGGGCCAGGGTTCTTACC |
| TNFα-R | GCCCACCCATGTCAAGTTCT |
| CCL5-F | GTGGGTGCGAGAGTACATCAAC |
| CCL5-R | GGCGCAAGTTCAGGTTCAAG |
| XCL1-F | TCATTACCAGACGTGGCCTTAAA |
| XCL1-R | TTGTTTGGACGGCTTTTTTCA |
| CXCL10-F | GGTCCTTAGAAAAACTTGAACTGATTC |
| CXCL10-R | TCCTTTTCATTGTGGCAATAATCTC |
| RPS24-F | AAGGAACGCAAGAACAGAATGAA |
| RPS24-R | TTTGCCAGCACCAACGTTG |
